# In low protein diets, microRNA-19b regulates urea synthesis by targeting SIRT5

**DOI:** 10.1038/srep33291

**Published:** 2016-09-30

**Authors:** Rui-Ping Sun, Qian-Yun Xi, Jia-Jie Sun, Xiao Cheng, Yan-Ling Zhu, Ding-Ze Ye, Ting Chen, Li-Min Wei, Rui-Song Ye, Qing-Yan Jiang, Yong-Liang Zhang

**Affiliations:** 1 College of Animal Science, Chinese National Centre of Pig Breeding Technology, ALLTECH-SCAU Animal Nutrition Control Research Alliance, National Engineering Research Center for Breeding Swine Industry, South China Agricultural University, 483 Wushan Road, Guangzhou, 510642, China; 2 Institute of Animal Science and Veterinary Medicine, Hainan Academy of Agricultural Science, Haikou 571100, China

## Abstract

Ammonia detoxification, which takes place via the hepatic urea cycle, is essential for nitrogen homeostasis and physiological well-being. It has been reported that a reduction in dietary protein reduces urea nitrogen. MicroRNAs (miRNAs) are major regulatory non-coding RNAs that have significant effects on several metabolic pathways; however, little is known on whether miRNAs regulate hepatic urea synthesis. The objective of this study was to assess the miRNA expression profile in a low protein diet and identify miRNAs involved in the regulation of the hepatic urea cycle using a porcine model. Weaned 28-days old piglets were fed a corn-soybean normal protein diet (NP) or a corn-soybean low protein diet (LP) for 30 d. Hepatic and blood samples were collected, and the miRNA expression profile was assessed by sequencing and qRT-PCR. Furthermore, we evaluated the possible role of miR-19b in urea synthesis regulation. There were 25 differentially expressed miRNAs between the NP and LP groups. Six of these miRNAs were predicted to be involved in urea cycle metabolism. MiR-19b negatively regulated urea synthesis by targeting SIRT5, which is a positive regulator of CPS1, the rate limiting enzyme in the urea cycle. Our study presented a novel explanation of ureagenesis regulation by miRNAs.

Nitrogen metabolism, which primarily involves urea and ammonia metabolism, is necessary for normal health. Ammonia detoxification is essential for physiological well-being in mammals^1^, and the hepatic urea cycle plays a predominant role in ammonia disposal, converting ammonia to urea for excretion^2^. Previous studies have shown that a reduction in dietary protein represents an effective and practical method of reducing urea nitrogen in swine^3^,^4^ humans^5^, and rats^6^,^7^. Dietary recommendations for patients with genetic hyperammonemia and urea cycle disorders include dietary protein restriction and supplementation with urea cycle substrates to limit ammonia synthesis and/or enhance ammonia excretion^5^,^8^,^9^. Additionally, studies have shown that the activity and expression of urea cycle enzymes in the liver vary based on nutritional perturbations^10^,^11^. In modern livestock production, reducing nitrogenous wastes, especially nitrous oxide and ammonia, is considered to be an important strategy for reducing environmental waste and improving productivity.

MicroRNAs (miRNAs) play a key role in the regulation of gene expression by modulating the stability and/or translation efficiency of target mRNAs^12^, a number of miRNAs have a post-transcriptional regulatory effect on genes involved in diverse biological processes including development^13^, differentiation^14^, cell proliferation^15^, cell cycle^16^, energy metabolism, fat metabolism^17^, and glucose homeostasis^18^. MiRNA levels in hepatocytes have been measured using deep sequencing methods^19^. Changes in hepatic miRNA profiles might reflect underlying liver injury or inflammation. MiR-122, which accounts for 70% of the total miRNAs in the liver, has been the target of extensive research due to its association with cholesterol metabolism and hepatocellular carcinoma and its role in promoting hepatitis C virus replication^20^,^21^. Only four miRNAs, i.e., miR-221-3p, miR-221-5p, miR-222-3p, and miR-326-3p, are induced by ammonia and promote ammonia-induced growth arrest and senescence in cultured rat astrocytes^22^. However, there is little information on the effect of miRNA on ureagenesis. Moreover, it is still unknown whether a low protein diet reduces urea synthesis via miRNA-mediated post-transcriptional regulation.

In our work, we found reduecd serum urea nitrogen (SUN) levels in weaned piglets with fed reduced CP. Proteomic analysis revealed that protein expression of CPS1 and SIRT5 was reduced (data not shown). Here, we measured miRNA expression in hepatic tissue of pigs fed different protein diets using Solexa/Illumina deep sequencing to obtain a comprehensive view to reveal their functions in urea metabolism and specifically to identify critical miRNAs that play key roles in the regulation of urea cycle under low protein diets.

## Results

### Differentially expressed hepatic miRNAs in LP group

Solexa/Illumina deep sequencing was used to determine the hepatic miRNA expression profile in the LP group. Unique sequences were identified based on mature miRNA sequences in miRBase (release 20.0), including 301 known pig miRNAs, and 116 miRNAs were mapped to the pig genome (Table S2). Among the 116 miRNAs, miR-122, a liver-specific miRNA expressed at high levels in human liver and adult mouse^23^,^24^, was the most abundant miRNA in piglets. A total of 755 novel miRNAs, labelled as PC-3p or PC-5p, were identified. In general, the novel miRNAs were expressed at very low levels. Only three miRNAs (PC-3p-391_4946, PC-5p-1261_1347, and PC-5p-1464_1159) had expression levels >300 RPM. As a result of their low expression levels, we eliminated most of the novel miRNAs from subsequent analyses.

**Figure 1:**
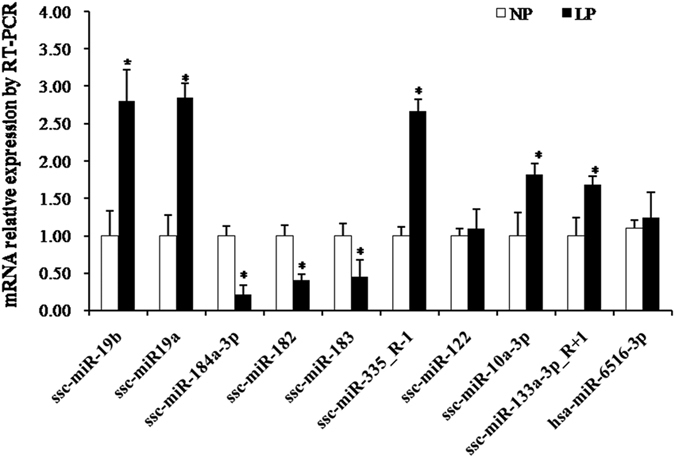
Validation of differentially expressed miRNAs by qPCR, Data are presented as mean ± SD (n = 6). *P < 0.05. LP: low protein die, NP: normal protein diet.

There were 25 differentially expressed miRNAs between the NP and LP groups; 19 were upregulated and six were downregulated with expression levels >1.5-fold (P < 0.05; Table S3). To validate the miRNA expression profile obtained from sequencing, we randomly selected 10 differentially expressed miRNAs for stem-loop RT-qPCR. With the exception of miR-6516-3p, nine miRNAs were successfully detected by RT-qPCR, and their expression profiles were consistent with those obtained from sequencing (Fig. 1). Compared to NP, LP had a higher expression of ssc-miR-19b (2.81-fold higher based on qRT-PCR and 1.64-fold higher based on sequencing).

### MiRNA target prediction and KEGG pathway analyses

**Figure 2:**
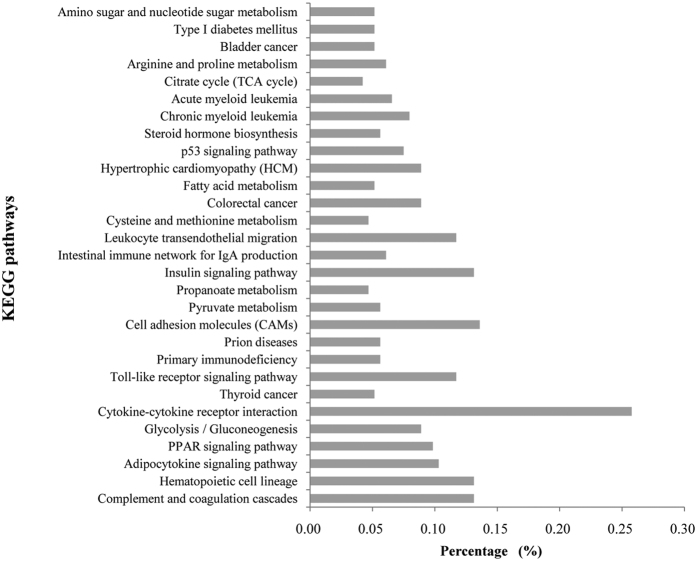
KEGG pathway analysis of most abundant miRNAs.

To better understand the roles of different miRNAs in piglet metabolism, potential targets of miRNAs were explored. Twenty-five different miRNAs were predicted to target 1,905 potential transcripts using RNA hybrid. KEGG pathway enrichment analysis coupled to DAVID functional annotation identified 27 enriched pathways (P < 0.05, Fig. 2; Table S4). Pathways involving arginine and proline, which regulate nitrogen metabolism, were enriched^25^.

### Targets of miRNAs in the urea cycle

**Figure 3:**
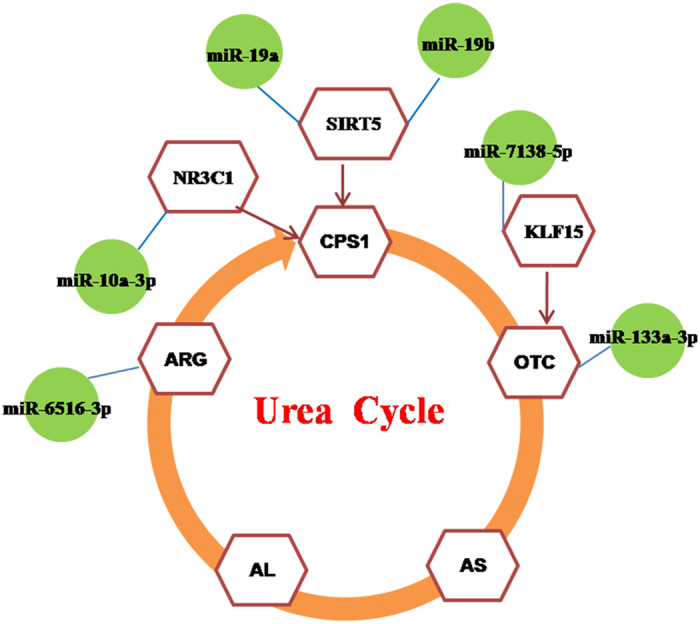
MiRNAs targeting the urea cycle network. The different miRNAs were analysed; six miRNAs participated in the urea cycle.

Six genes, i.e., OTC, ARG1, SIRT5, NRC31, K1F15, and OAT, play important roles in urea metabolism^25^,^26^,^27^,^28^,^29^. To better understand the interactions between miRNAs and their target genes, a miRNA-mRNA network was constructed (Fig. 3). Interestingly, miRNAs-targeted genes involved in the urea cycle were upregulated in the LP group compared to the NP group.

### Urea nitrogen analysis and expression of SIRT5 and CPS1 in LP and NP groups

**Figure 4:**
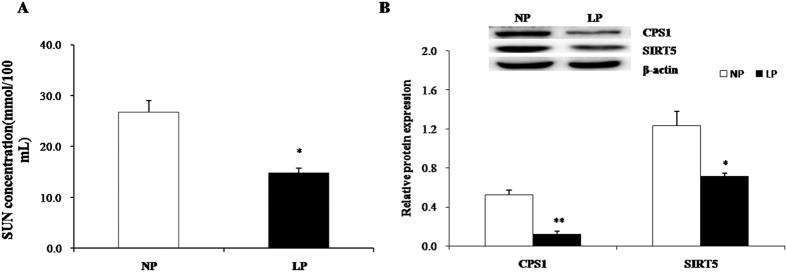
Low protein diet induces urea synthesis in weaned piglet liver. (**A**) Serum urea nitrogen; (**B**) CPS1 and SIRT5 protein expression. Data are presented as mean ± SD. (*P < 0.05, **P < 0.01, n = 6). LP: low protein die, NP: normal protein diet.

A reduction in CP contributed to low urea levels in serum and liver (Fig. 4A) as previously reported^30^,^31^,^32^,^33^. Moreover, CPS1 protein expression levels (data not shown) were significantly reduced in LP compared to NP. Western blot results revealed that SIRT5 and CPS1 expression levels were lower in LP than in NP (Fig. 4B).

### Target prediction of miR-19b and verification by using luciferase report assay

**Figure 5:**
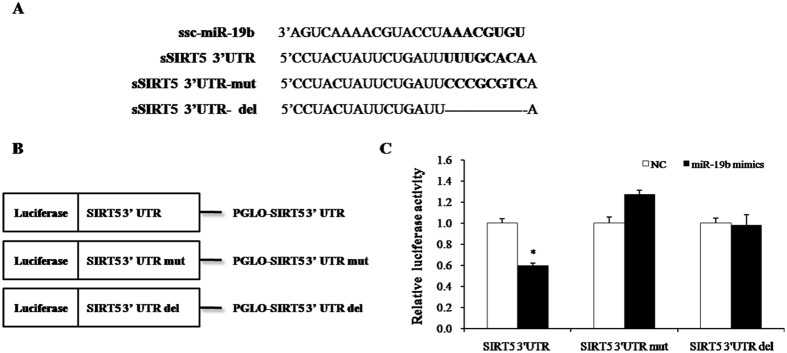
MiR-19b directly targets SIRT5. (**A**) Three 3′-UTR sequences containing normal, mutagenic, or deleted binding sites were inserted downstream of the luciferase reporter. Eight nucleotides of SIRT5 3′ UTR were mutated or deleted to disrupt the binding with miR-19b seed regions. (**B**) Schematic diagram showing dual-luciferase reporter constructs of pig SIRT5 3′ UTR with putative miR-19b-3p binding site. (**C**) Constructed vectors were transfected into CHO cells with NC or miR-19b mimic. The luciferase assay results revealed significant differences between NC and miR-19b-mimic groups transfected with vectors containing normal SIRT5 3′-UTR (*P < 0.05, n = 8).

To verify the target relationship between ssc-miR-19b and 3′-UTR of porcine SIRT5 (Fig. 5A), the full-length sequence 3′-UTR of SIRT5 was inserted into the pmirGLO vector (Promega) to construct the recombinant Dual-Luciferase reporter vector pGLO-SIRT5-3′UTR. Additionally, the seed sequence was either mutated or deleted to disrupt the miR-19b binding site (Fig. 5B). The wild-type (pGLO-SIRT5 3′-UTR) or mutant (pGLO-SIRT5 3′-UTR-mut and pGLO-SIRT5 3′-UTR-del) plasmid were co-transfected with an miR-19b mimic into CHO cells. As shown in Fig. 5C, the luciferase activity of the wild-type SIRT5 reporter was significantly reduced (P < 0.05) by ssc-miR-19b mimic compared with the negative control at 48 h post-transfection. The reduction was rescued both by mutation and deletion of the seed sequence (Fig. 5C).

### Identification of porcine primary hepatocytes

To identify the isolated porcine primary hepatocytes, cellular morphology was observed. The isolated cells in cell suspension in a single free decentralized state and cell body is round and bright (Fig. S1A), After incubation for 4 h, hepatocyte readily attached onto the plate surface (Fig. S1B), whereas other cells remained suspended in the medium and were eliminated by rinsing with culture medium. As the culture progressed from 24 h to 48 h, hepatocyte showed obvious morphology changes with a typical polygonal morphology with round nuclei, between cells shaped Island connection (Fig. S1C,D). These results were in according with early reports^28^,^34^ and showed it feasible for further experiments.

### Confirmation of urea synthesis regulation by miR-19b in porcine primary hepatocytes

**Figure 6:**
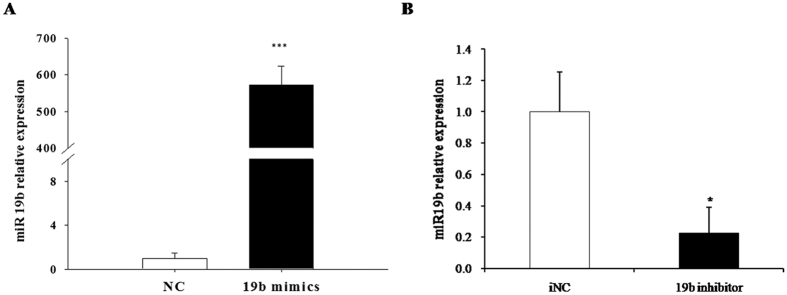
Transfection with miR-19b mimic and inhibitor. Pig primary hepatocytes were treated with miR-19b mimic, miR-19b inhibitor, or their corresponding negative controls. MiR-19b expression in pig primary hepatocytes was measured by qRT-PCR after 48 h post-transfection. (**A,B**) Data are presented as mean ± SD (*P < 0.05, ***P < 0.001, n = 6).

We evaluated the effectiveness of miR-19b mimic throughout the experimental process by transfecting it in porcine primary hepatocytes. Compared with the control group, miR-19b levels dramatically increased at 48 h post-transfection (Fig. 6A). In contrast, miR-19b expression levels were notably reduced by inhibitor in porcine primary hepatocytes (P < 0.05) relative to iNC (Fig. 6B). These results suggested that miR-19b mimic was feasible for further experiments.

**Figure 7:**
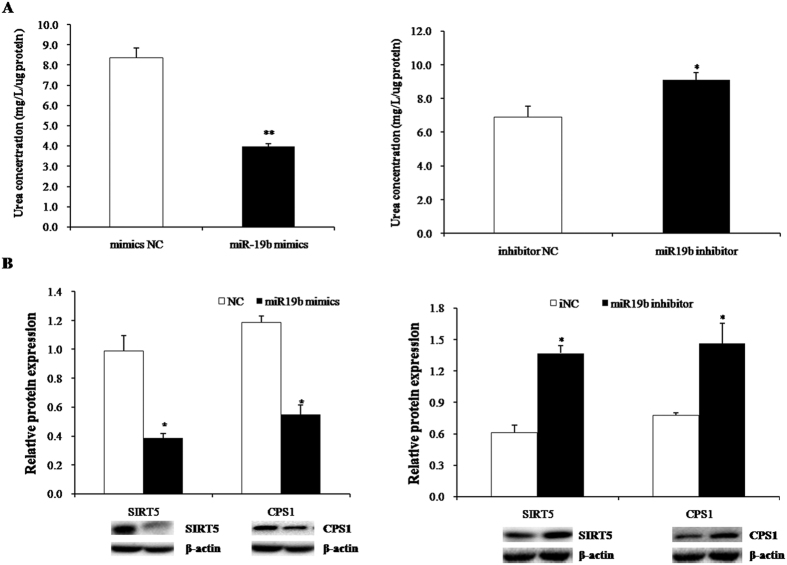
MiR-19b regulates ureagenesis in pig primary hepatocytes. (**A**) Urea concentration in hepatocytes treated with miR-19b mimic, inhibitor, or negative control. Data are presented as mean ± SD (*P < 0.05, **P < 0.01, n = 6). (**B**) Western blot results showed that SIRT5 protein and CPS1 protein expression in primary hepatocytes was significantly decreased by miR-19b mimic versus control; miR-19b inhibitor rescued this effect. Data are presented as mean ± SD (*P < 0.05, n = 6).

To gain further insight on the role of miR-19b in hepatic urea synthesis, urea production of porcine primary hepatocytes was measured at 48 h post-transfection with miR-19b mimic, inhibitor, or NC. As shown in Fig. 7, porcine primary hepatocytes with miR-19b mimic had a significant reduction in urea nitrogen levels, and miR-19b inhibitor rescued this reduction (Fig. 7A,B). Western blotting results showed that miR-19b mimic decreased SIRT5 and CPS1 protein levels (P < 0.05, Fig. 7C), and the effect was rescued by miR-19b inhibitor (Fig. 7D). The results revealed that the reduction in SIRT5 and CPS1 activity was mediated by miR-19b, which was correlated with changes in urea synthesis.

**Figure 8:**
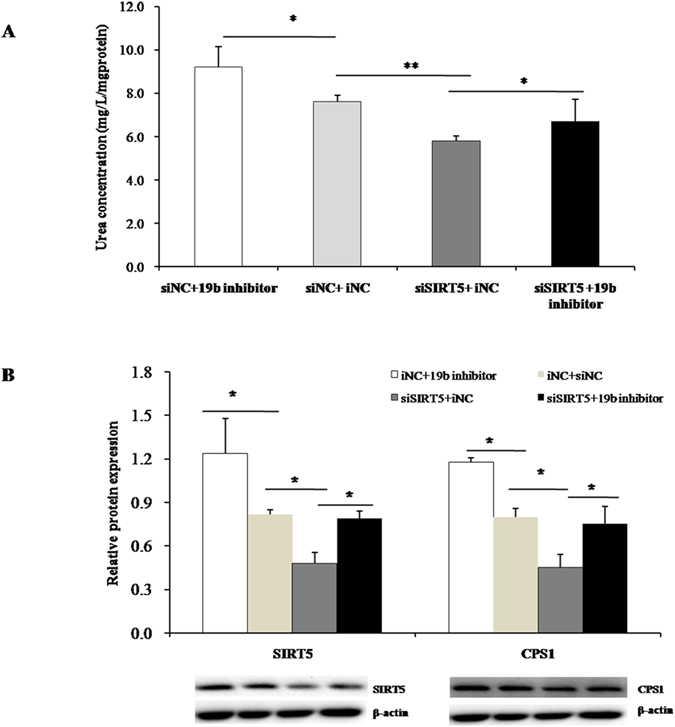
Combination trial of miR-inhibitor and SIRT5 siRNA Primary hepatocytes co-transfected with *SIRT5* siRNA and miR-19b inhibitor or inhibitor negative control, (**A**) urea concentration in hepatocytes supernatant was assayed by urea nitrogen assay kit. Data are presented as mean ± SD (*P < 0.05, **P < 0.01,n = 6); (**B**) The protein expression of SIRT5 and CPS1 were simultaneously detected by western blotting. Data are presented as mean ± SD (*P < 0.05, n = 6).

To further examine the relationship between miR-19b and SIRT5, we performed co-transfection experiments with miR-19b inhibitors or control oligos and siSIRT5 or non-specific control siRNA. Knockdown SIRT5 significantly decreased urea synthesis; therefore, SIRT5 inhibition had similar effects to those observed with miR-19b overexpression (Fig. 8A). Interestingly, this effect was rescued with the addition of miR-19b inhibitor (inhibitor + siSIRT5). Western blotting results further confirmed the direct regulation of miR-19b on SIRT5 (Fig. 8B). The miR-19b inhibitor rescued the inhibition of siRNA on SIRT5; therefore, it is most likely that miR-19b directly regulated SIRT5.

## Discussion

In piglets, SUN levels decreased with low dietary CP levels, as previously reported^30^,^31^,^32^,^33^. The urea cycle converts ammonia into water-soluble and non-toxic urea and plays an important role in the regulation of acid-base balance in mammals. In several species, including pigs, blood urea nitrogen (BUN) can be used to quantify nitrogen utilization and excretion rate^35^. High BUN concentrations indicate low dietary protein or amino acid utilization^36^. Additionally, BUN concentrations are indicative of chronic kidney diseases and/or liver disorders^37^,^38^.

There were 25 differentially expressed hepatic miRNAs between the low and normal protein groups. Among these miRNAs, six candidate miRNAs, i.e., ssc-miR-19a, ssc-miR-19b, ssc-miR-10a-3p, ssc-miR-7138-5p, ssc-miR-133a-3p, and hsa-miR-6516-3p, play a role in the urea cycle. Interestingly, these six miRNAs were upregulated in the low protein group. Additionally, a significant upregulation of miR 19b was observed in the low protein group as quantified by qPCR. To our knowledge, this is the first study that identifies miRNAs associated with hepatic urea synthesis.

MiR-19b belongs to the miR-17/92 cluster, which plays a role is cancer pathogenesis, cell proliferation, liver regeneration, and other diseases^39^,^40^,^41^. Studies have revealed that miR-19 has the main oncogenic effects of the entire cluster^42^. Specifically, miR-19b may be involved in the pathogenesis of cardiovascular diseases^43^, neuroinflammation^44^, colon carcinoma^45^, and adipocyte differentiation^46^; however, little was known about the role of miR-19b in urea metabolism. This study provides the first evidence that miR 19b has a regulatory role in urea synthesis.

Bioinformatics predicted that miR-19b binds to the 3′UTR of SIRT5. The luciferase assay confirmed that miR-19b directly targeted the seed sequence in SIRT5 3′-UTR, because both miR-19b mimic and mutation/deletion of the seed sequence decreased luciferase activity. Furthermore, Western blotting results revealed that SIRT5 protein levels were reduced with the overexpression of miR-19b and that SIRT5 protein levels increased when miR-19b was inhibited.

SIRT5 is a unique member of the Sirtuin family with multiple functions in cellular metabolism^47^. SIRT5 deacetylates and activates CPS1^48^, the rate-limiting enzyme in hepatic ureagenesis^49^. Decreased ureagenesis in low protein diets in mammals is associated with a reduction in CPS1 gene expression in the liver. A recent report has shown that upregulation of CPS1 and deacetylation of CPS1 by SIRT5 are observed in high protein diets and that blood ammonia in SIRT5 knockout mice is significantly higher than in wild-type mice following a 48-h fast^28^,^50^. Additionally, CPS1 activity and urea production in Sirt5-Tg mice are higher than in wild-type mice^48^. Our results showed that a reduction in SIRT5 protein expression by miR-19b mimic resulted in the downregulation of CPS1 with a concomitant reduction in urea synthesis in pig primary hepatocytes. The miR-19b inhibitor had opposite effects to those obtained with miR-19b mimic. Furthermore, the use of siRNA against SIRT5 confirmed the relationship between miR-19b and SIRT5, because siRNA interferes with gene expression^51^. Results have shown that miR-19b inhibitor rescued the downregulation of SIRT5 and CPS1 and the siRNA-induced reduction of urea, which provided further evidence on the direct action of miR-19b on SIRT5.

Disorders in the urea cycle cause life-threatening hyperammonemia^52^, and miRNA replacement might be an effective therapy^53^. Additionally, miRNAs have been evaluated as diagnostic markers of liver diseases^54^. Therefore, miR 19b may be a useful therapeutic and diagnostic agent in urea cycle disorders.

In summary, miRNA expression was affected in piglets consuming a low protein diet, and miR-19b inhibited ureagenesis by targeting SIRT5. Our study highlighted a novel explanation of ureagenesis regulation by miRNA and provided a novel strategy for ureagenesis regulation in humans and animals.

## Methods

### Ethics statement

The animals were handled in strict compliance with the Welfare and Ethics of Laboratory Animals Regulations established by the Chinese Association for Laboratory Animal Sciences. All animal procedures were performed according to the protocol SCAU-AEC-2010-0416, which was approved by the Institution of Animal Ethics Committee of South China Agricultural University.

### Animals and samples

The feeding trial was performed at the Institute of Subtropical Agriculture of The Chinese Academy of Science (Changsha, China) as previously reported^55^. Briefly, a total of 12 weaned piglets (Duroc × Landrace × Yorkshire) were acquired from the experimental field of the animal observation station in South China Agricultural University. The piglets (28 d of age), with an initial body weight (BW) of 9.57 ± 0.64 kg, were randomly assigned to one of two groups with six animals per group: a normal (20%) crude protein (NP) diet (n = 6) and a low (17%) crude protein (LP) diet (n = 6). The diets, which met the National Research Council (NRC; 2012) nutrient specifications for 11–20 kg BW pigs (Table 1), were based on corn-soybean and provided 14.6 MJ/kg digestible energy. Throughout the feeding trial, the piglets had ad libitum access to feed and water. Prior to the start of the feeding trial, the piglets underwent a seven-day adaptation period to the new diets. At the end of the feeding trial (30 d), blood samples (approximately 10 ml) were collected from each pig by venepuncture, and the piglets were immediately euthanized. Following centrifugation of blood samples at 3,000 × g, the supernatant (serum) was transferred into tubes and frozen in liquid nitrogen. Livers were aseptically excised, rinsed in ice-cold saline, and stored in liquid nitrogen while being transported to the laboratory, where they were stored at −80 °C.

### Urea nitrogen analysis

The primary hepatocyte were seeded in 6-well plates at density of 1 × 10^6^ per well. After transfection with the miR-19b mimic/inhibitor and NC/iNC, the cells supernatant were collected for urea nitrogen assay. Urea nitrogen (UN) of serum and cell supernatant were measured using a commercial kit (C0132, NanJingJianCheng Bioengineering Institute, NanJing, China) based on the urease Berthelot colorimetry method. The urea were quantified using the internal standard urea by measuring the absorption of reaction products at 640 nm. Cells total protein was detected using the BCA Protein Detection Kit (Bioteke Corporation, Beijing, China) for normalization of cell urea concentration.

### Total RNA isolation

Total RNA was extracted using Trizol reagent (Invitrogen, CA, USA) and treated with DNase to eliminate trace genomic DNA. Total RNA integrity was determined by examining the 28S and 18S rRNA bands on ethidium bromide-stained agarose gels. Total RNA quantity and purity were assessed in a Bioanalyzer 2100 and RNA 6000 Nano LabChip Kit (Agilent, CA, USA).

### Construction and sequencing of small RNA libraries

Two libraries were generated from the total RNA extracted from the LP and NP group. Data processing was performed as previously reported^56^. Briefly, raw reads were subjected to an Illumina pipeline filter (Solexa 0.3) to obtain mappable sequences using ACGT101-4.2 (LC Sciences, Hangzhou, China) based on mammalian data in miRBase 20.0. Modified reads per million reads (RPM) were used to quantify the normalized reads. The unique sequences were achieved and used for subsequent analysis. All the sequence data have been submitted to the NCBI Gene Expression Omnibus (http://www.ncbi.nlm.nih.gov/sra/) under accession NO. SRP074399.

### Real-time quantitative PCR for miRNAs and mRNA

Reverse transcription was performed with 1 μg total RNA using M-MLV Reverse Transcriptase and a specific hairpin primer for the miRNA (Table S1)^57^. Real-time quantitative PCR (RT-qPCR) was performed in a Bio-Rad CFX-96 real-time PCR thermocycler (Bio-Rad, USA) using miScript II RT and miScript SYBR Green PCR kits (Qiagen) with a miRNA-specific forward primer (Table S1) and a universal reverse primer. The PCR reaction (20 μl) consisted of 2 μl of cDNA, 1.5 μM of each primer, 10 μl of 2× SYBR Green PCR Master Mix (Toyobo Co., Ltd., Osaka, Japan), and distilled water. The reactions were processed for 1 min at 94 °C, followed by 40 cycles of 15 s at 95 °C, 15 s at 58 °C, and 40 s at 72 °C. MiRNA expression level was normalized to that of the internal control U6 in each sample. Expression was subsequently standardized to the miRNA by the 2^−ΔΔCt^ method.

### Target prediction and Kyoto Encyclopedia of Genes and Genomes pathway analyses

Target prediction and Kyoto Encyclopedia of Genes and Genomes (KEGG) pathway analyses were performed. Porcine miRNA targets were obtained at the whole pig genome level (sscrofa10.2, www.ensembl.org/ Sus_scrofa/)^58^. KEGG pathway analyses were performed using DAVID bioinformatics resources (http://david.abcc.ncifcrf.gov/).

### Western blot analysis

Hepatic homogenates were prepared by homogenizing frozen livers in cold RIPA buffer containing protease inhibitors (Boston Bio Products, MA). Hepatic protein (20 μg) was subjected to NuPAGE 5–10% Bis-Tris gel electrophoresis and transferred to polyvinylidene fluoride membranes (0.45 μm), which were subsequently blocked with 5% milk, incubated overnight at 4 °C with primary antibodies against CPS1 (Santa Cruz Biotechnology, Santa Cruz, CA) and SIRT5 (Cell Signaling Technology, Inc., Danvers, USA) followed by secondary antibodies for 1 h at room temperature. β-actin (Abcam, Cambridge, UK) was used as the control. Duplicate experiments were carried out for all hepatic proteins. The blot was scanned in a FluorChem M (ProteinSimple, Santa Clara, California, USA). The data were analysed by Image J software and expressed as fold-change relative to the control group after normalizing against β-actin.

### Cell culture

Five-day-old neonatal pig (Landrace, SCAU, Guangzhou, China) livers were perfused, and hepatocytes were isolated and purified using a two-step procedure^59^,^60^. Briefly, pigs were fasted for 12 h and euthanized with sodium pentobarbital injection (50 mg/kg). The livers were initially perfused via the inferior vena cava with phosphate buffered saline (PBS, pH 7.2, devoid of calcium and magnesium) containing 10 mM 4-(2-hydroxyethyl)-1-piCperazineethanesulfonic acid (HEPES) and 5 mM ethylenediaminetetraacetic acid to remove blood cells and hydrolysed with 0.4 mg/ml collagenase IV (SIGMA, China, C125 collagen digestion units per mg) in PBS buffered with 10 mM HEPES. The perfused livers were passed through a 400-mesh sieve, and the hepatocytes were collected following centrifugation at 800 × g for 5 min at 4 °C. After washing the cells twice, isolated hepatocytes were seeded into 6-well culture plates at a density of 1 × 10^5^ cells/cm^2^ in Williams’ E culture medium (SIGMA, China) supplemented with 10% fetal bovine serum (FBS, GIBCO, China), 1% SITE liquid media (containing 1.0 mg/ml bovine pancreas insulin, 0.55 mg/ml iron-free human transferrin, 0.5 μg/ml sodium selenite, and 0.2 mg/ml ethanolamine), 10 μg/ml glucagon, 10 nM dexamethasone (SIGMA, China), and 100 U/ml antibiotic (SIGMA, China). Cells were cultured on collagen-coated plates at 37 °C in a 5% CO_2_ incubator. After 4 h, the medium was replaced with fresh Williams’ E culture medium containing 10% FBS and 100 U/ml antibiotic, and the plates were incubated for 24–36 h. Chinese hamster ovary (CHO) cells were maintained in Roswell Park Memorial Institute medium 1640 (GIBCO) supplemented with 10% FBS.

### Plasmid construction

The 3′-UTR sequences of porcine transcripts in whole genome were obtained from NCBI (http://www.ncbi.nlm.nih.gov/). The 3-UTR of SIRT5 (Accession NO.: EU357901.1) contains the highly conserved binding sites for the miR19b, and the sequence 486bp is as Table S5, Further, the 3′-UTR sequence was inserted into pmirGLO Vector (Promega) with XhoI and XbaI double digestion to construct recombinant Dual-Luciferase reporter vector, named as pGLO-SIRT5 -3′UTR (Fig. 1A). Meanwhile, Mutagenic and deleted SIRT5 3′UTR reporter vectors Table S5, i.e., pGLO-SIRT5-3′UTR-mut and pGLO-SIRT5-3UTR-del, respectively, were constructed with seven exchanged nucleotides or a deleted target site via DNA synthesis (Sangon Biotech, Shanghai).

### Luciferase reporter assay

CHO cells were seeded at density of 3 × 10^4^ cells per well in 96-well plates. When the cells reached 60–70% confluency, wild-type (pGLO-SIRT5 3′-UTR) or mutant (pGLO-SIRT5 3′-UTR-mut and -del) plasmid was co-transfected with miR-19b mimic or negative control (NC) into CHO cells. Lipofectamine 2000 (Invitrogen) was used for mediating the transfection. Cells were collected 48 h post-transfection, and the luciferase assay was performed with the Dual-Luciferase reporter assay system (Promega), the luciferase activity was normalized by renilla activity. Statistical differences between treatment and control groups were determined using Student’s t-test at P < 0.05.

### MiR-19b mimic, miR-19b inhibitor, and siRNA for SIRT5

MiR-19b mimic, miR-19b inhibitor, negative control (NC and iNC), and siRNA for porcine SIRT5 with siNC were acquired from Shanghai GenePharma Co. When the pig primary hepatocytes reached 60–70% confluency, miR-19b mimic or inhibitor (100 pmol) was transfected into the cells. NC and iNC were used as negative controls for the miR-19b mimic and miR-19b inhibitor, respectively. Transfection was performed using Lipofectamine 2000 (Invitrogen). SiSIRT5/siNC was co-transfected with miR-19b inhibitor/iNC. Following 48 h, cell supernatants were collected and stored at −80 °C for subsequent measurements of urea nitrogen. Hepatocytes were rinsed twice with PBS and stored at −80 °C.

### Statistical analysis

Data were presented as mean ± SD. Data analysis was performed with Student’s t-test or one-way ANOVA using SPSS17.0 (SPSS Inc., Chicago, IL). P < 0.05 was considered to be statistically significant and was denoted by an asterisk, P < 0.01 was denoted by two asterisks, and P < 0.001 was denoted by three asterisks.

## Additional Information

**How to cite this article**: Sun, R.-P. *et al*. In low protein diets, microRNA-19b regulates urea synthesis by targeting SIRT5. *Sci. Rep.* **6**, 33291; doi: 10.1038/srep33291 (2016).

## Supplementary Material

Supplementary Information

Supplementary Table S1

Supplementary Table S2

Supplementary Table S3

Supplementary Table S4

## Figures and Tables

**Table 1:** Composition (g/kg) and nutritional value of diets^a^.

Ingredients	Content (%)^b^	
	17.00	20.00
Corn	66.50	63.70
Soybean meal	18.80	19.80
Dried whey	4.30	4.30
fish meal	4.00	9.00
Soybean oil	2.60	0.80
L-lysine	0.62	0.38
DL-methionine	0.19	0.10
L-threonine	0.21	0.09
DL-tryptophan	0.04	0.01
Calcium hydrophosphate	0.74	0.00
Limestone	0.70	0.52
Salt	0.30	0.30
1% premix compound^c^	1.00	1.00
Total	100.00	100.00
**Calculated nutrient content**		
DE (MJ/kg)	14.60	14.60
CP	17.00	20.00
Total Ca	0.71	0.69
Total P	0.55	0.57
Lys	1.23	1.23
Met + Cys	0.68	0.68
Thr	0.73	0.73
Trp	0.20	0.20
Arg	0.91	1.09
His	0.40	0.46
Ile	0.60	0.70
Leu	1.32	1.49
Phe	0.69	0.80
Val	0.65	0.77
EAA/NEAA	0.80	0.70
Recommendation rate of NRC (2012)	0.80	0.80

^a^Diets contain 17%, and 20% CP, respectively, with appropriate crystalline AA supplementation.

^b^The values are expressed as percentage (%), except for DE (MJ/kg), EAA/NEAA, and recommendation rate of NRC(2012).

^c^Premix provided these amounts of vitamins and minerals per kilogram on an as-fed basis: vitamin A, 10 800 IU; vitamin D3, 4000 IU; vitamin E, 40 IU; vitamin K3, 4 mg; vitamin B1, 6 mg; vitamin B2, 12 mg; vitamin B6, 6 mg; vitamin B12, 0.05 mg; biotin, 0.2 mg; folic acid, 2 mg; niacin, 50 mg; D-calcium pantothenate, 25 mg; Fe, 100 mg as ferrous sulfate; Cu, 150 mg as copper sulphate; Mn, 40 mg as manganese oxide; Zn, 100 mg as zinc oxide; I, 0.5 mg as potassium iodide; and Se, 0.3 mg as sodium selenite.
