# Supplementary material for: In low protein diets, microRNA-19b regulates urea synthesis by targeting SIRT5
Source: Sci Rep. 2016 Sep 30;6:33291. doi: 10.1038/srep33291 (PMC5043173; doi:10.1038/srep33291)
Supplement: Supplementary Information [file srep33291-s1.doc]

**Supplementary information file**

**In low protein diets, microRNA-19b regulates urea synthesis by targeting SIRT5**

**Rui-Ping Sun, ^1^,** ^2,#^ **Qian-Yun Xi ^1^,** ^#^**, Jia-Jie Sun^1^, Xiao Cheng, ^1^ Yan-Ling Zhu ^1^ , Ding-Ze Ye, ^1^ Ting Chen, ^1^ Li-Min Wei, ^1^,**^2^ **Rui-Song Ye, ^1^ Qing-Yan Jiang, ^1^ Yong-Liang Zhang^1^ ***

^1^College of Animal Science, Chinese National Centre of Pig Breeding Technology, ALLTECH-SCAU Animal Nutrition Control Research Alliance, National Engineering Research Center for Breeding Swine Industry, South China Agricultural University, 483 Wushan Road, Guangzhou, China, 510642.

^2^ Institute of Animal Science and Veterinary Medicine, Hainan Academy of Agricultural Science, Haikou 571100, China

^#^ These two authors contributed equally to this paper

***** Corresponding author: [zhangyl@scau.edu.cn](mailto:zhangyl@scau.edu.cn)

**Supplemental Figure Legends**

**Fig S1 .** Primary culture of pig hepatocytes after 0h(A), 4 h (B), 24h(C) and 48h(D) of culture.


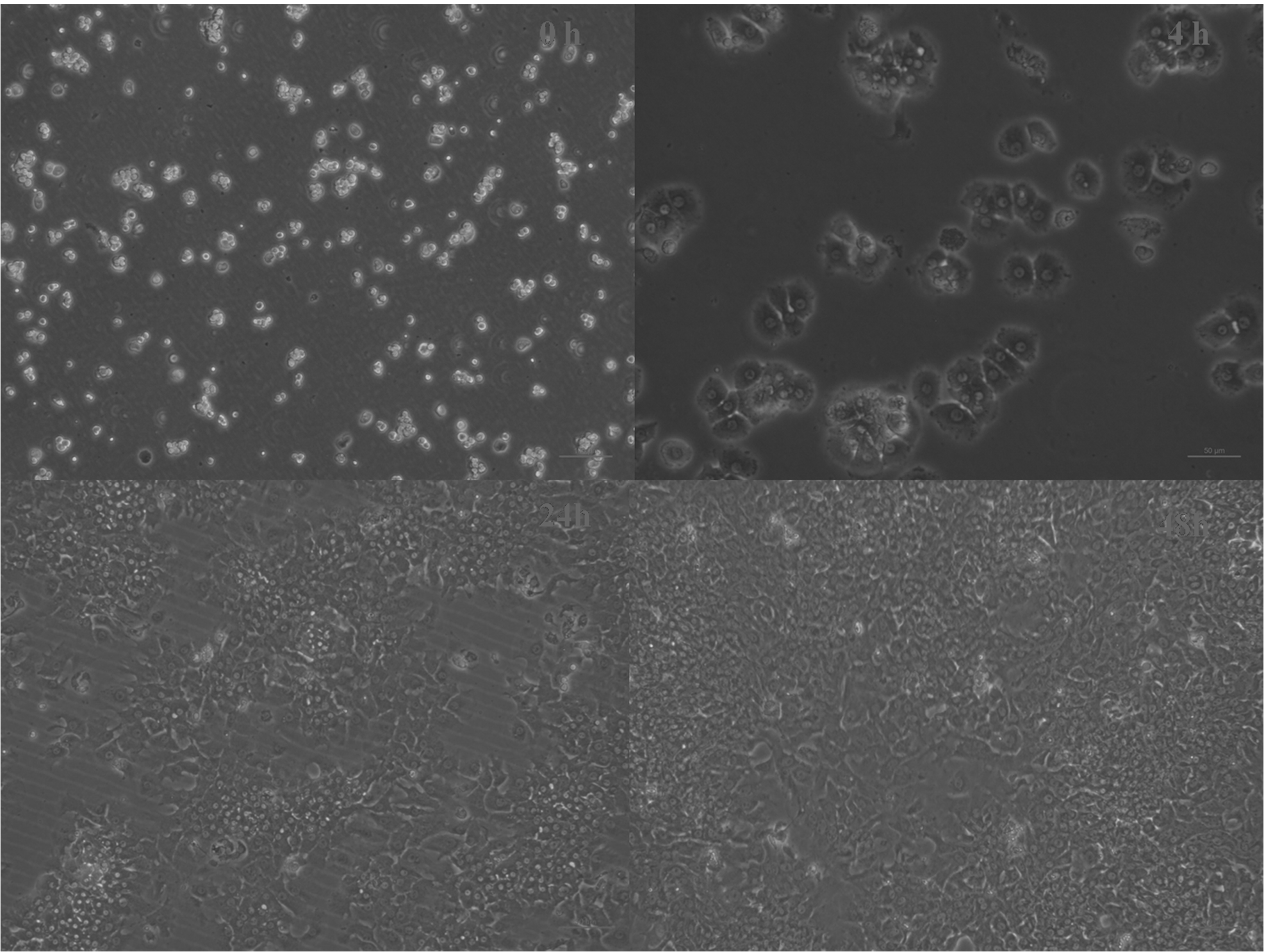
**Figure S1**

**Table s5 sequence of SIRT5 plasmid vector**

| **Sequence of SIRT5 -3’UTR(486 bp)** |
| --- |
| GCTCTAGATGAAACTGAACCTGTCTCTTAGTCGTCCCGGGGAAGGAAGAGATCACAGCACACCGAAGTACCAGGCCACCGACGGGAGGAGCTGCCTTGCCGATGGTGAGCTGGAGGTTGGAAGGTCTGGCAACATCTTGTGATTCTCCGGTTGGAATCCTCACTGCTGCCAAGTGACAGGGACGTAGAAGCAGGGGGAGTAAAGGTGTTCGGAAGTCATAGAACTTCTGAAGTCAATTCATGGGATTTGGTTTGAACCGAAACACAAGGTGCCTCTGACACGTTTGGTTGGTGACGGGGAGGGACACAAGTTTGTCAGGAAATGATCTAAAAGACGTAATTTTCCGGATTGTATTTCTGCTCTTTGGGCAAGGCTAGAAGTAGAGGGCTAAAGCCCTGGTATTTGTGACTTTTGCACAAGGTTTGGTGGAAAATAAAAGCACTCTCTACAGCCGGTCATTTGCTGTATAAAGATGGTTCTCGAGGG |
| **Sequence of SIRT5 -3’UTR-mut(161 bp)** |
| GGAAATGATCTAAAAGACGTAATTTTCCGGATTGTATTTCTGCTCTTTGGGCAAGGCTAGAAGTAGAGGGCTAAAGCCCTGGTATTTGTGACTCCCGCGTCAGGTTTGGTGGAAAATAAAAGCACTCTCTACAGCCGGTCATTTGCTGTATAAAGATGGTT |
| **Sequence of SIRT5 -3’UTR-del(154 bp)** |
| GGAAATGATCTAAAAGACGTAATTTTCCGGATTGTATTTCTGCTCTTTGGGCAAGGCTAGAAGTAGAGGGCTAAAGCCCTGGTATTTGTGACTAAGGTTTGGTGGAAAATAAAAGCACTCTCTACAGCCGGTCATTTGCTGTATAAAGATGGTT |
